# Mandibular Alveolar Ridge Split With Simultaneous Implant Placement: A Case Report

**DOI:** 10.7759/cureus.31156

**Published:** 2022-11-06

**Authors:** Kamal Prasad Pandey, Radhika Sunil Kherdekar, Hoshang Advani, Seema Dixit, Ashutosh Dixit

**Affiliations:** 1 Dentistry (Periodontics), All India Institute of Medical Sciences, Rishikesh, Rishikesh, IND; 2 Dentistry (Conservative & Endodontics), Seema Dental College, Rishikesh, IND

**Keywords:** digital impression, piezosurgical unit, ridge split, bone atrophy, dental implant

## Abstract

Bone resorption following tooth loss is an obvious, continuous, and unpredictable process, which poses one of the greatest challenges in implant placement. The posterior regions of the jaws show more resorption compared to the anterior regions, with the mandible being affected more. Augmentation of the narrow alveolar ridge has been done using various techniques. The alveolar ridge split technique (ARST) is frequently used for the horizontal augmentation of the narrow ridge. In this case report, a 47-year-old female patient who had partial edentulism on the lower left jaw region associated with a narrow alveolar ridge was treated using the ridge split technique. A piezosurgical unit was used for splitting the ridge, followed by simultaneous implant placement. This alveolar ridge split technique is considered to be more predictable, reliable, and successful as compared to other techniques such as autogenous onlay bone graft and guided bone regeneration.

## Introduction

Ridge resorption after teeth loss is an obvious, unpredictable, and continuous process. Most changes around the alveolus occur as a result of extraction, which leads to alteration in the width and height of the ridge. This usually happens due to the normal remodeling of bone following extraction, traumatic extraction, periodontal disease, surgical resection, prolonged denture wear, or disuse atrophy. The loss of a tooth can cause significant ridge resorption in all three planes, the most prominent being in the horizontal direction [1].

Such narrow, atrophic alveolar ridges pose a challenge and greater difficulty for successful restoratively driven implant placement as a tooth replacement option. We can place dental implants only if there is adequate bone to stabilize them, and there should be a minimum of 1 to 1.5 mm of bone all around the implant for successful osseointegration [2].

Several methods have been implicated to augment the narrow alveolar ridge, such as guided bone regeneration (GBR) using various graft materials (autograft, allograft, xenograft, and alloplast), autogenous onlay block grafts harvested intra-orally or extra-orally, distraction osteogenesis, ridge expansion osteotomy, and ridge splitting [3].

Ridge expansion by means of hand osteotomes with subsequently increasing dimensions was first invented by Tatum [4] and then modified by Summers [5]. The alveolar ridge split technique (ARST) involves splitting the buccal or labial and lingual or palatal cortical tables and gradually opening the space, which heals in a manner similar to an extraction socket [6].

Few anatomical requirements for successful ARST have been mentioned in the literature. These include a minimum 2 mm horizontal bone width to ensure adequate cortical and cancellous bone on either side of the split ridge. There should be a minimum of 10 mm of bone height from the vital structures, absence of any concavity defect in the alveolar bone profile and osteotomies to be placed at least 1 mm away from the adjacent teeth [7].

There are several advantages of the alveolar ridge split technique such as the possibility of simultaneous implant placement, avoiding the requirement of secondary donor sites for bone graft harvesting, reduction in treatment time, and less patient morbidity [8].

Ridge split technique was initially performed with a chisel and hammer [9] or by using rotating and oscillating saws [10]. The use of a bone chisel can be stressful and traumatizing to the patient. Rotating and oscillating instruments are more likely to increase the risk of trauma to the gingiva, mucosa, lips, and tongue. Piezoelectric bone surgery (PEBS) presents a novel and atraumatic alternative technique for bone surgery. Piezosurgery is unique as it has the ability to cut only hard tissues, such as bone and teeth, while preserving the soft tissues such as gingiva, mucosa, blood vessels, nerves, and sinus membrane from the injury [11].

## Case presentation

This case report shows a horizontal ridge augmentation by the ridge split technique using a piezosurgical unit with simultaneous implant placement for partial edentulous lower arch rehabilitation.

A 47-year-old female patient visited the department of dentistry, All India Institute of Medical Science (AIIMS), Rishikesh, India, with the complaint of dislodged fixed partial denture on the lower left back region. The patient was a nonsmoker with no significant medical history. Teeth in the mandibular left posterior region were lost eight years back followed by replacement with a tooth-supported fixed partial denture.

**Figure 1:**
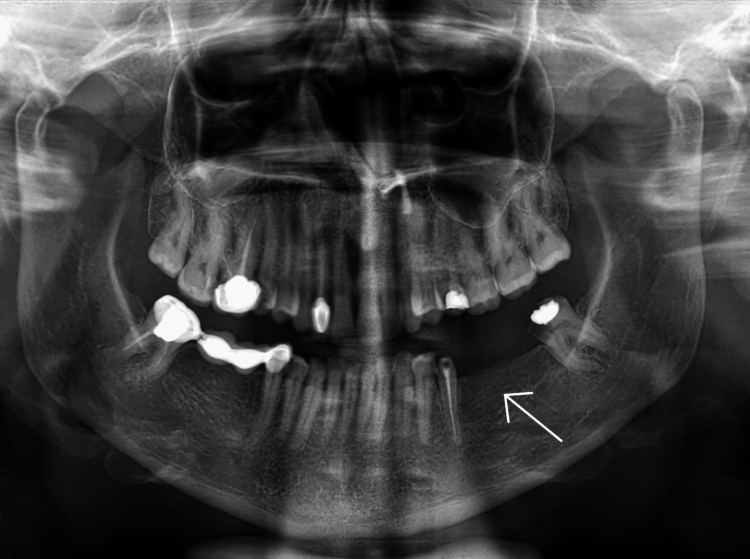
Preoperative panoramic radiograph

On clinical examination, the mandibular left first and second molars were missing. The second premolar was endodontically treated and the third molar on the same side was extensively decayed (Figure 1).

**Figure 2:**
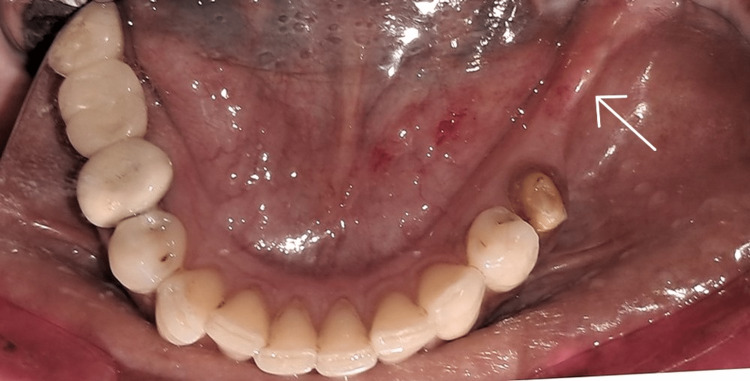
Initial clinical picture of the alveolar ridge

The non-restorable status of the third molar warranted extraction. Prosthodontic options were discussed with the patient, and she opted for a treatment plan that would allow for an implant-supported prosthesis. When the edentulous ridge was clinically examined, it was found to be resorbed and to have less buccolingual width (Figure 2).

**Figure 3:**
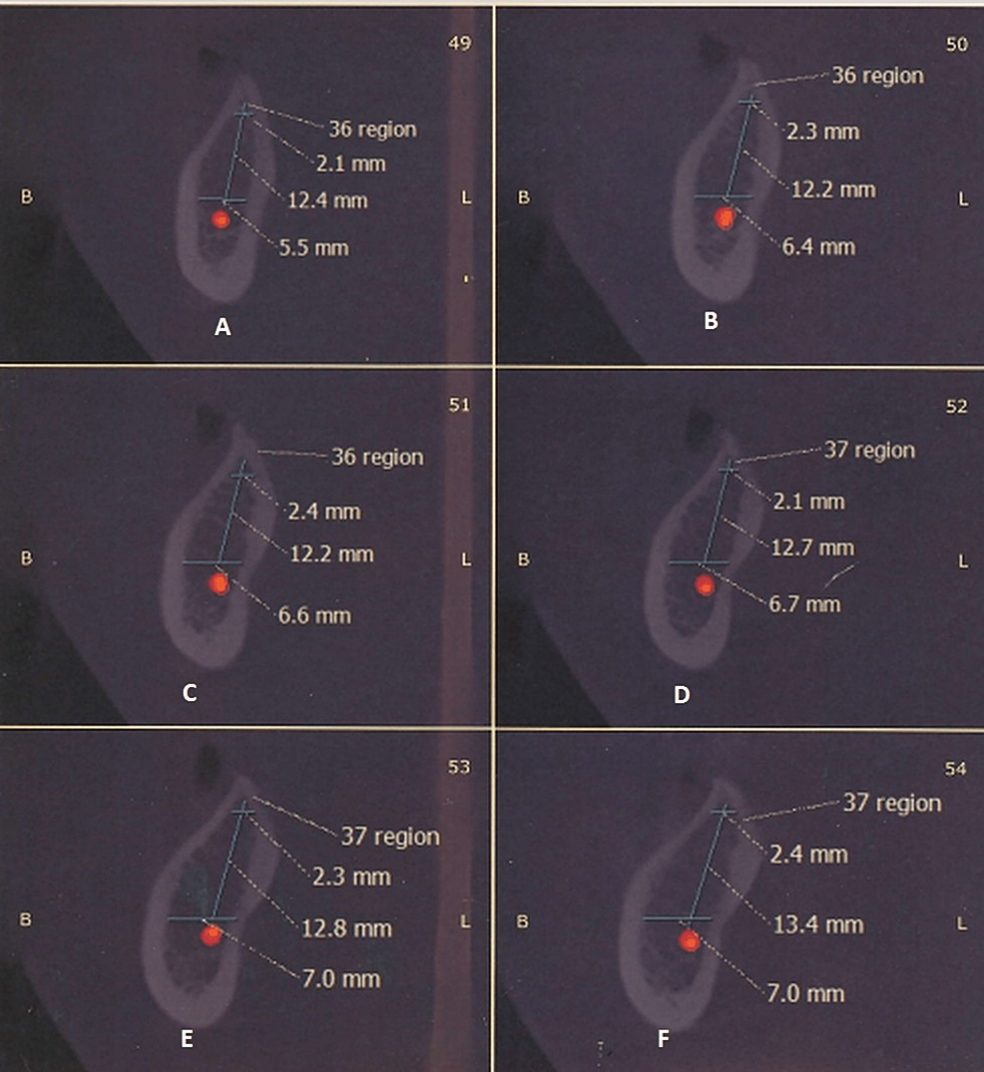
CBCT scans showing the measurements of bone ridge thickness A, B, and C show the 36 region, and D, E, and F show the 37 region CBCT: cone-beam computed tomography

The patient was advised cone-beam computed tomography (CBCT), and it showed that the width of the alveolar ridge was 2 to 2.5 mm in the crestal area of the ridge with progressive widening in the apical direction, which was inadequate for implant placement. However, the height from the inferior alveolar nerve to the alveolar crest was 12.5 mm, which was adequate (Figure 3).

All required routine blood investigations were found to be within the normal reference range, deeming the patient fit for undergoing the surgical procedure. An alveolar ridge split with simultaneous implant placement was planned with respect to the 36 and 37 regions.

Surgical procedure

**Figure 4:**
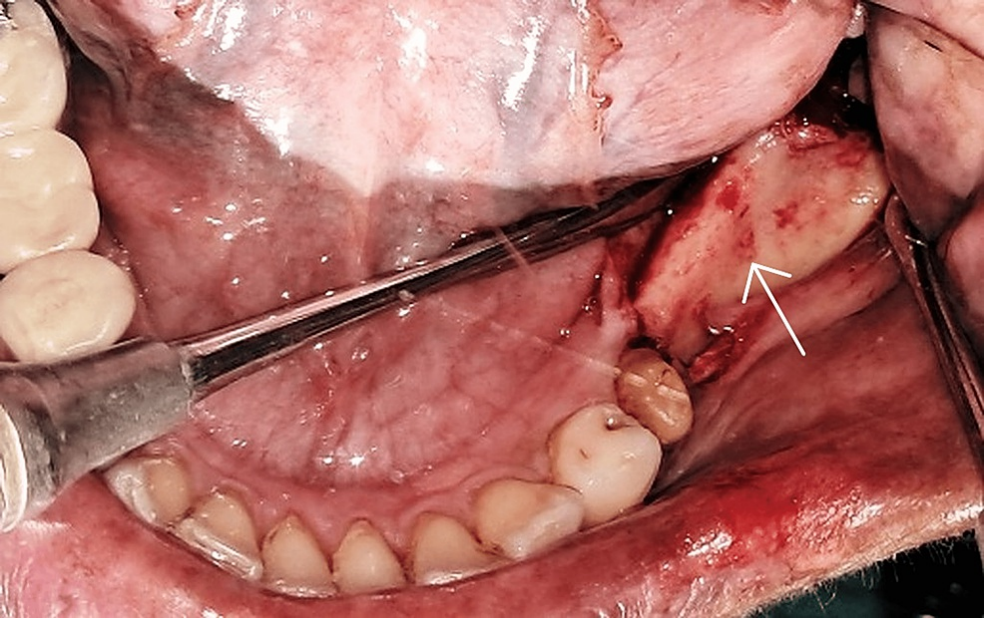
Full thickness flap elevated showing the narrow ridge

The patient was prepared for implant surgery according to the standard surgical protocol. Following the pre-surgical oral rinse with 0.2% chlorhexidine for 1 minute, the mandibular left region was anesthetized using 2% lignocaine with 1:100,000 epinephrine. A sharp mid-crestal incision along with mesial and distal vertical releasing incisions was placed, and a full-thickness mucoperiosteal flap was raised (Figure 4).

**Figure 5:**
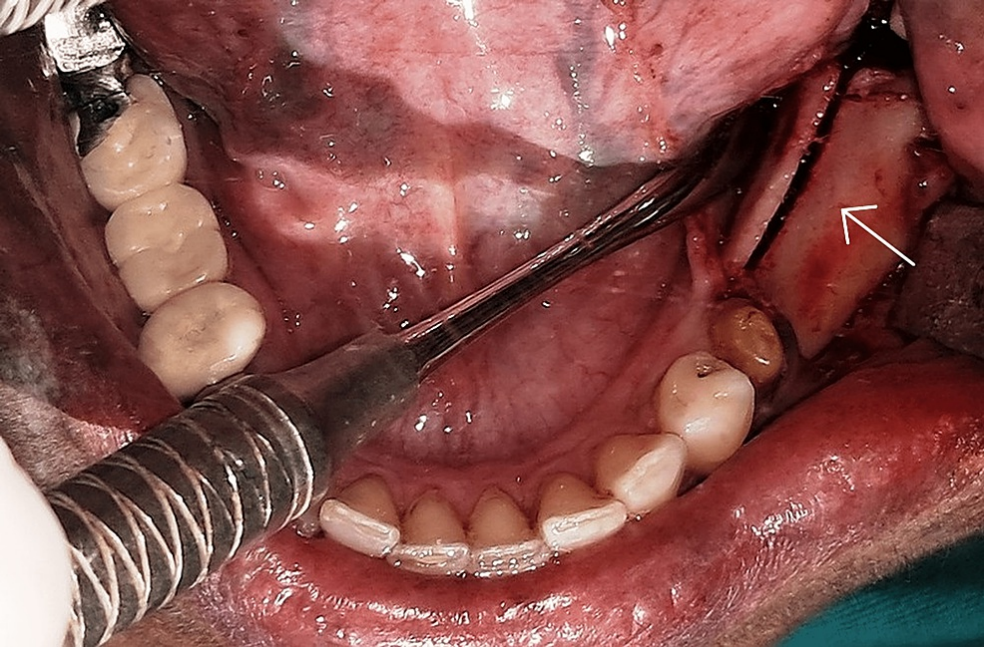
Post ridge split

A Piezosurgery unit (Acteon, England) was used for preparing the osteotomy in the center of the crest of the ridge, extending anteroposteriorly, 2 mm distal to the second premolar. The depth of the osteotomy was kept at 8 mm. Two vertical osteotomies were carried out in the mesial and distal ends of the mid-crestal osteotomy on the buccal cortical plate extending from the outer cortical bone to the inner cancellous bone. A small chisel and tapered osteotome were inserted in the mid-crestal osteotomy to create a green-stick fracture and a ridge split was created carefully (Figure 5).

**Figure 6:**
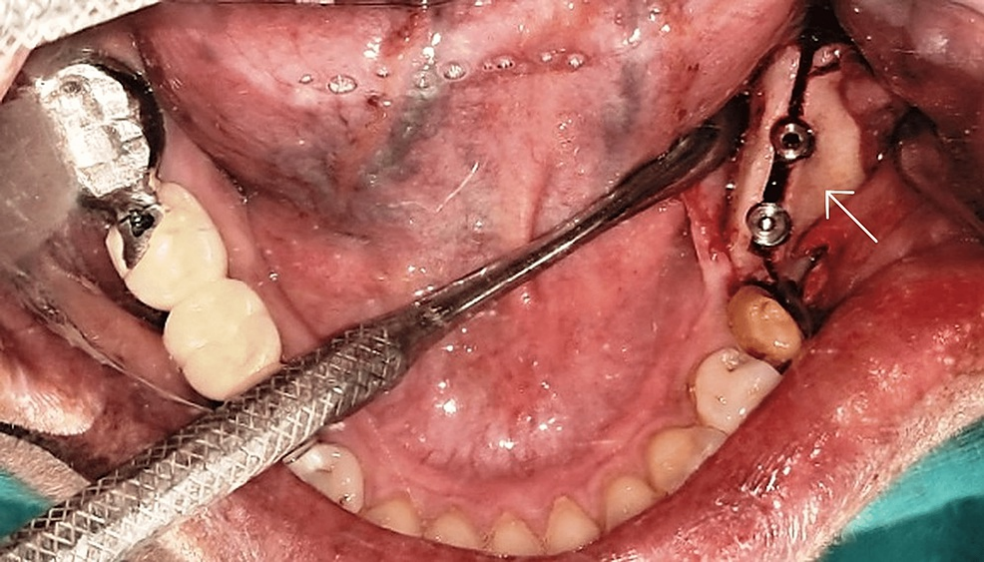
Two implants placed

Sequential implant osteotomies were prepared and two spiral dental implants (Bioline, Berlin, Germany) (3.75 mm diameter x 11.5 mm length) were subsequently placed in the molar region (Figure 6).

This was followed by the placement of bone xenograft (Osseograft, Advanced Biotech Products, Chennai, India) to fill the remaining expanded space. A periosteal releasing incision was given to extend the flap coronally for a tension-free close approximation. Interrupted suturing was done with 3.0 Silk (Ethicon Mersilk, Ethicon Inc., Raritan, New Jersey). Postoperative instructions were given to the patient. A non-steroidal anti-inflammatory drug (400 mg ibuprofen) and an anti-biotic (500 mg amoxicillin + 125 mg clavulanic acid combination) were directed to be taken three times a day for five days. The patient was advised to use 0.2% chlorhexidine oral rinse twice daily for 15 days.

**Figure 7:**
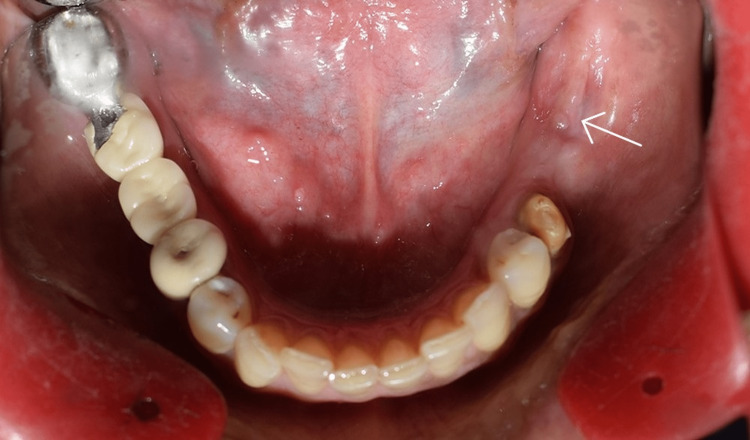
Four-month postoperative clinical picture of the alveolar ridge

The sutures were removed after 10 days. Healing was uneventful. Clinical and radiographic evaluations were performed after four months to assess the healing and changes in marginal bone level (Figure7).

**Figure 8:**
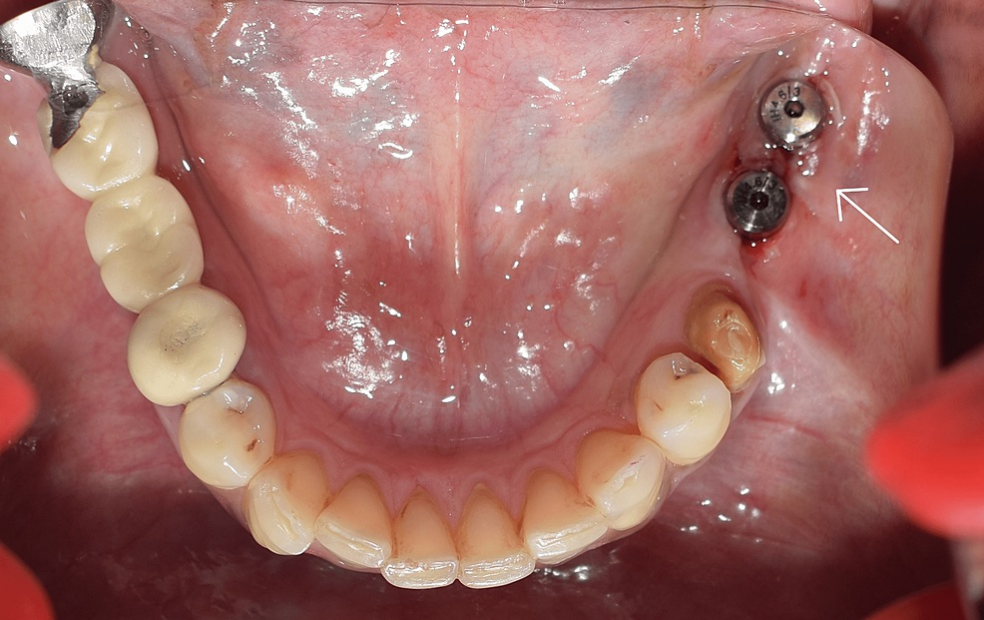
Healing abutments

There were no reported complaints or complications during this healing period. The implants were uncovered and 3 mm healing abutments were placed (Figure 8).

**Figure 9:**
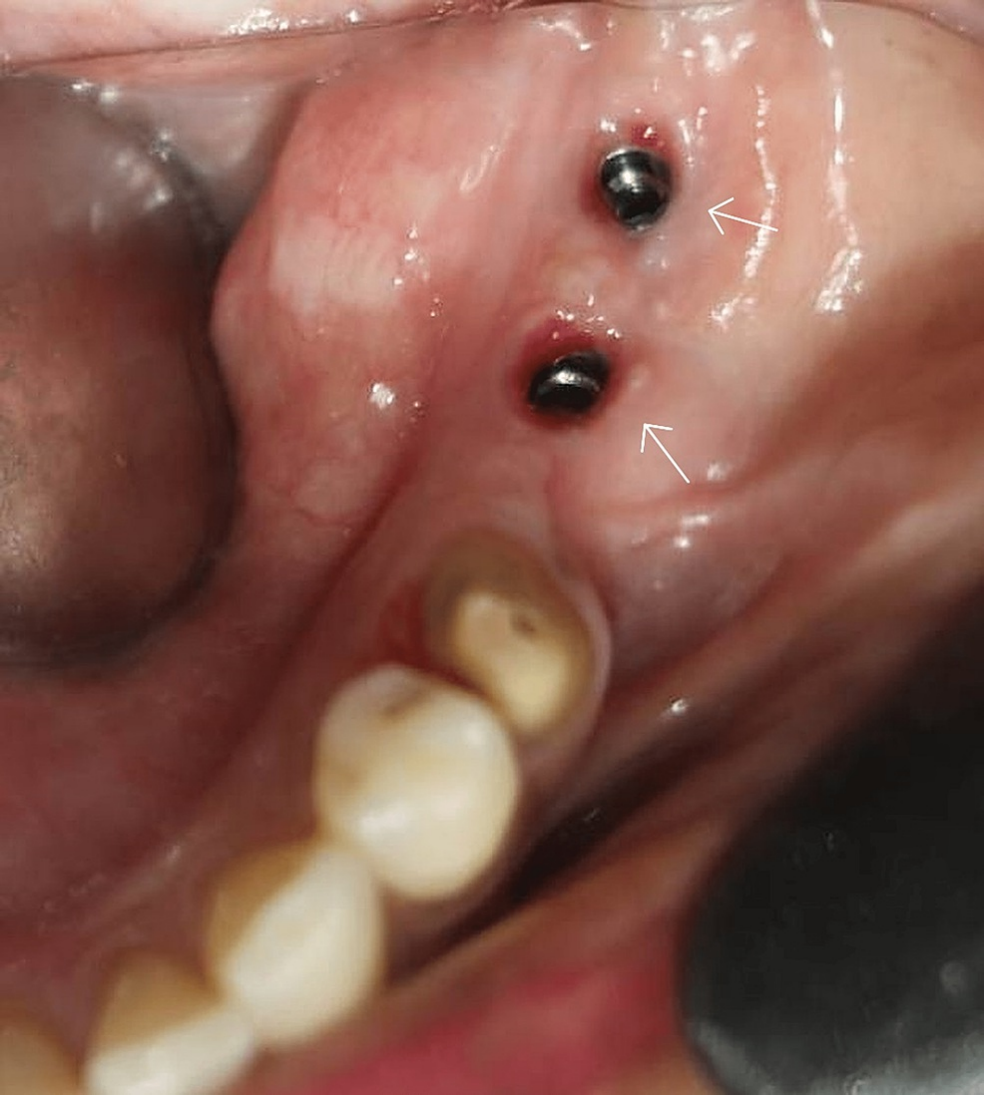
Gingival collar after healing abutment placement

Figure 9 shows the clinical picture after three weeks of tissue molding with the healing abutments.

**Figure 10:**
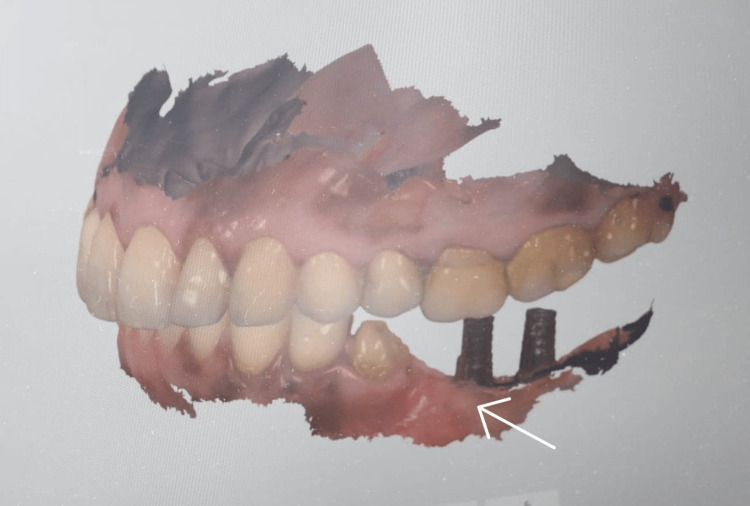
Digital implant impression

 The patient was recalled. the implant abutment was connected, and an abutment-level digital implant impression was made (Figure 10).

**Figure 11:**
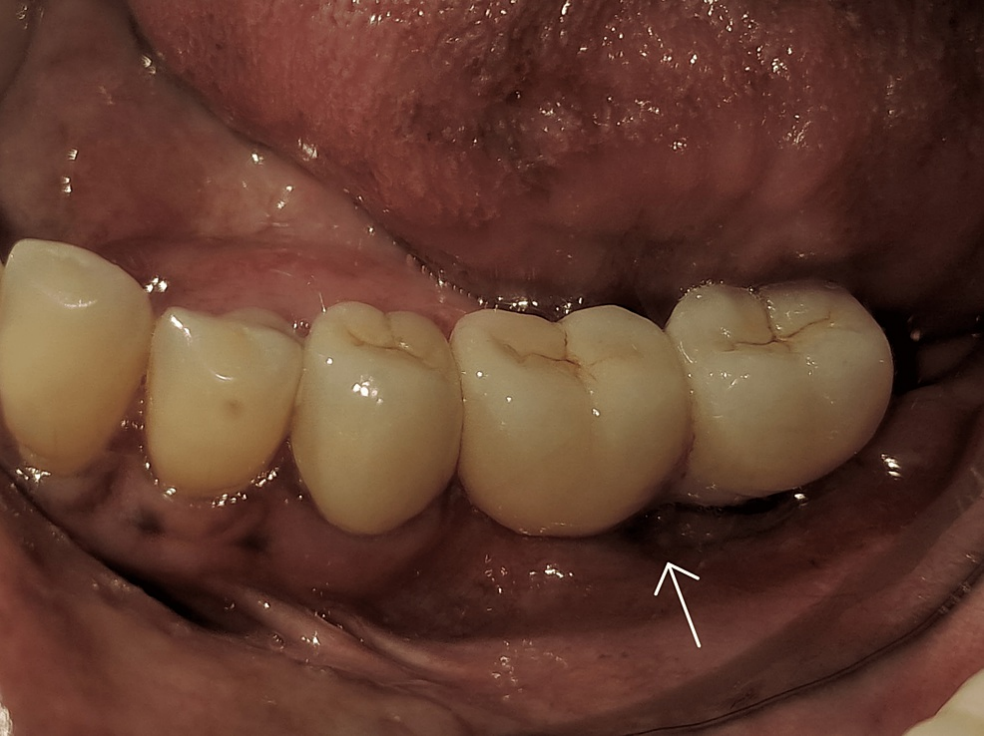
Final prosthesis placed

The implant-supported and splinted ceramic prosthesis was cemented a week later (Figure 11).

**Figure 12:**
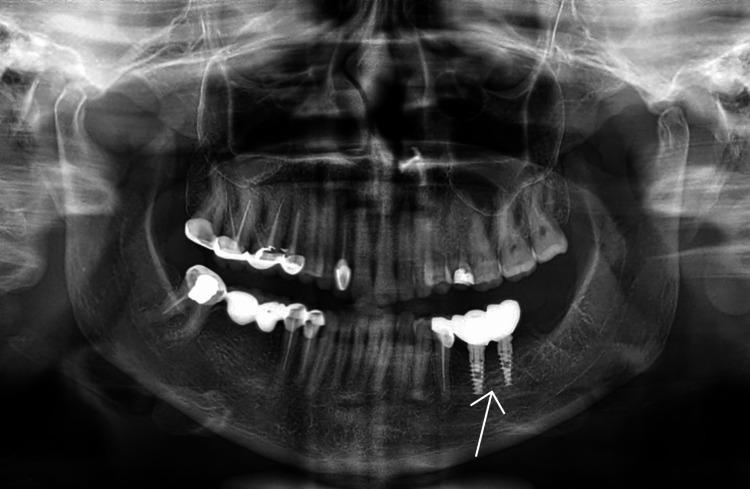
Postoperative OPG OPG: orthopantomagram

**Figure 13:**
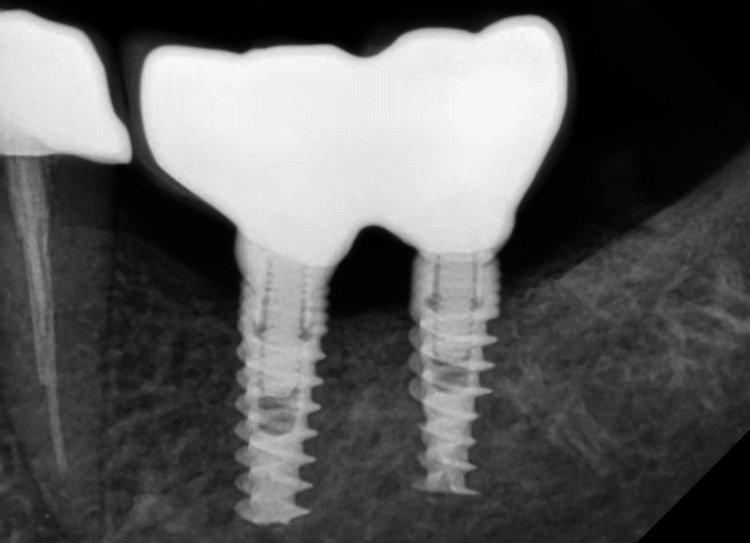
Postoperative IOPA IOPA: intraoral periapical radiograph

A postoperative X-ray was done (Figures 12, 13).

## Discussion

The main goal of dental implant therapy is to replace missing teeth in order to achieve oral rehabilitation. Narrow alveolar ridges are accompanied by difficulty in placement and less favorable prosthetic outcomes and, thus, should be augmented prior to implant placement for obtaining a successful result [1]. For lateral ridge augmentation, onlay bone grafts have been used. However, these techniques require more invasive surgical procedures and a second surgical donor site to obtain the graft [12]. In addition, the grafted site is left to heal for a period of five to seven months, and further implant placement and its osseointegration prolong the treatment duration immensely. Studies indicate that the rate of lateral onlay bone graft resorption ranges from 20 to 50% after seven months. Mandibular sites showed a greater amount of bone graft resorption than maxillary sites [13]. For GBR procedures, the drawbacks include the collapse of the membrane, resorption of grafting material, and exposure of the membrane, leading to infection. This too involves long healing periods with unpredictable bone formation [14].

The ridge split technique offers the advantages of being less invasive and producing more predictable outcomes. Simion et al., in their original surgical procedures, found that the aim of the ridge split was to create a ‘‘self-space-making’’ defect that allowed for better containment of graft in the space created by the surrounding bony walls [15]. A greenstick fracture can be made on the buccal cortical plate to displace it in a labial direction, and this expanded space heals in a similar manner to that of an extraction socket [10]. The greatest advantage of this procedure is eliminating the need for a second surgical site and its associated morbidity, and simultaneous implant placement thereby reducing treatment time and cost-effectiveness and increasing dental implant stability [8]. Ridge splitting can also be done in a staged approach where a risk of cortical fracture is envisioned. Implant placement can be delayed, which allows mature bone growth and ensures better primary stability of implants [16].

It has been found that ARST has a higher success rate when performed on the maxillary arch compared to the mandibular arch [17]. A previous systematic review by Elnayef B et al. found a gain in horizontal bone width of approximately 4.13 ± 3.13 mm following ARST [8]. The D3 and D4 bone is pliable and contain a greater amount of cancellous bone, which allows extensive displacement of cortical plates with minimum risk of fracture. According to a systematic review by Mestas G, survival rates of titanium implants following ARST were 96% at 58 months [18].

In this case, the resorbed narrow alveolar ridge was bound anteriorly with teeth. The posterior mandibular site, along with the healing extraction socket of the mandibular left third molar, provided conducive conditions to carry out ridge splitting with the least chance of fracture of the cortical plate.

Traditionally, osseous surgery has been performed by either manual or motor-driven instruments. With the introduction and development of piezoelectric bone surgery, the limitations of conventional bone-cutting instruments have been minimized. Unique properties of piezosurgery, such as micro-precision, selective cutting, maximum visibility, and excellent healing, favor better results than traditional methods, and patient discomfort is also minimized. Attempting to create a space between the buccal and lingual cortical plates with traditional drills increases the risk of fracture of the dense buccal cortical plate. Thus, piezoelectric surgery has greatly simplified the ridge splitting and expansion technique [11].

In this case, a digital impression was obtained by the use of intraoral scanners (IOSs) with a fully digital workflow that scans the dental hard and soft tissue using projecting light. Reproducible hard and soft tissues are shown on the hardware display as natural looking. Intraoral cameras use photo techniques or video techniques for intra-oral scanning. Computer-aided design/computer-aided manufacturing (CAD/CAM) systems were used for the design and fabrication of prostheses. Digital impression (DI) has several advantages such as the lowered risk of distortion during impression making and model fabrication, shortening of the chairside time, and increased patient comfort [19].

As with any other technique, ARTs also have a few shortcomings. Some of these include: it is an operator-dependent technique with a gradual learning curve, it can only be used for horizontal bone gain, there is no vertical bone gain, and its result is better only for D2, D3, and D4 types of bone. This technique also poses greater challenges when used for ridge expansion in a single missing tooth due to the lack of bone elasticity and the chance of implant loss due to improper osseointegration [17]. In contrast, ARTs are associated with fewer technical complications by approximately 6.8% [20].

## Conclusions

The ridge-splitting technique provides the advantage of ridge expansion and simultaneous implant placement for the management of a narrow alveolar ridge. Proper patient evaluation and case selection are crucial for achieving successful surgical and prosthetic outcomes. Digital impression and CAD/CAM design prostheses provide more successful outcomes.
